# Strain- and Subtype-Specific Replication of Genotype 3 Hepatitis E Viruses in Mongolian Gerbils

**DOI:** 10.3390/v16101605

**Published:** 2024-10-12

**Authors:** Tiancheng Li, Yusuke Sakai, Yasushi Ami, Yuriko Suzaki, Masanori Isogawa

**Affiliations:** 1 Department of Virology II, National Institute of Infectious Diseases, Tokyo 208-0011, Japan; nisogawa@niid.go.jp; 2 Department of Pathology, National Institute of Infectious Diseases, Tokyo 208-0011, Japan; sakaiyu@niid.go.jp; 3 Division of Experimental Animals Research, National Institute of Infectious Diseases, Tokyo 208-0011, Japan; yami@niid.go.jp (Y.A.); ysuzaki@niid.go.jp (Y.S.)

**Keywords:** genotype 3 hepatitis E virus, HEV-3, subtype, Mongolia gerbil, small-animal model

## Abstract

Since Mongolian gerbils are broadly susceptible to hepatitis E virus (HEV), including genotypes 1, 4, 5, and 8 (HEV-1, HEV-5, HEV-5, and HEV-8) and rat HEV, they are a useful small animal model for HEV. However, we have observed that the subtypes HEV-3k and HEV-3ra in genotype 3 HEV (HEV-3) were not infected efficiently in the gerbils. A small-animal model for HEV-3 is also needed since HEV-3 is responsible for major zoonotic HEV infections. To investigate whether gerbils can be used as animal models for other subtypes of HEV-3, we injected gerbils with five HEV-3 subtypes (HEV-3b, -3e, -3f, -3k, and -3ra) and compared the infectivity of the subtypes. We detected viral RNA in the gerbils’ feces. High titers of anti-HEV IgG antibodies in serum were induced in all HEV-3b/ch-, HEV-3f-, and HEV-3e-injected gerbils. Especially, the HEV-3e-injected animals released high levels of viruses into their feces for an extended period. The virus replication was limited in the HEV-3b/wb-injected and HEV-3k-injected groups. Although viral RNA was detected in HEV-3ra-injected gerbils, the copy numbers in fecal specimens were low; no antibodies were detected in the sera. These results indicate that although HEV-3′s infectivity in gerbils depends on the subtype and strain, Mongolian gerbils have potential as a small-animal model for HEV-3. A further comparison of HEV-3e with different genotype strains (HEV-4i and HEV-5) and different genera (rat HEV) revealed different ALT elevations among the strains, and liver damage occurred in HEV-4i- and HEV-5-infected but not HEV-3e- or rat HEV-infected gerbils, demonstrating variable pathogenicity across HEVs from different genera and genotypes in Mongolian gerbils. HEV-4i- and HEV-5-infected Mongolian gerbils might be candidate animal models to examine HEV’s pathogenicity.

## 1. Introduction

Hepatitis E virus (HEV), classified in the family *Hepeviridae*, possesses a single-stranded, positive-sense RNA genome, and HEV causes type E hepatitis in humans [1,2]. The *Hepeviridae* family includes two subfamilies: *Orthohepevirinae* and *Parahepevirinae* [2,3]. *Parahepevirinae* consists exclusively of the genus *Piscihepevirus*, and the viruses in this genus infect only fish as a non-pathogenic fish virus [3,4]. *Orthohepevirinae* includes at least four genera: *Paslahepevirus*; *Rocahepevirus*; *Chirohepevirus*; and *Avihepevirus* (ictv.global/taxonomy) [3]. The genus *Paslahepevirus* is comprised of two species, *alci* and *balayani*. The species *balayani* includes at least eight genotypes, from HEV-1 to HEV-8, and it forms major HEV strains that are pathogenic to humans [3]. HEV-1 and HEV-2 are found exclusively in humans. HEV-3 and HEV-4 have been isolated from both humans and animals, including monkeys, domestic pigs, wild boars, wild deer, rabbits, and mongooses. HEV-5 and HEV-6 are detected in wild boars. HEV-7 and HEV-8 are detected in camels [2,3,4]. HEV-1, -2, -3, -4, and -7 infect humans and cause hepatitis E, whereas HEV-5 and HEV-8 infect monkeys and have the potential for zoonotic infection [5,6,7]. Rat HEV (genus *Rocahepevirus*) is also known to transmit to monkeys and humans and cause hepatitis E [8,9,10].

Cell culture systems have been established to grow HEVs that belong to *Paslahepevirus* (HEV-1, HEV-3 to HEV-8) and *Rocahepevirus* (rat HEV and ferret HEV) in PLC/PRF/5 cells [6,7,11,12,13,14,15]. Most HEVs experimentally cross-infect cynomolgus and rhesus monkeys, and these monkeys are useful animal models for HEV infection and vaccine development [10,16,17,18]. However, most imported cynomolgus monkeys and farmed rhesus have been exposed to HEV [19,20], and the supply of these monkeys for infection experiments is, thus, limited. Animal models are needed for HEV research, and small-animal models are desired.

Rabbits and rats have been used as the respective animal models of HEV-3ra, HEV-4, or rat HEV; however, most HEV strains, such as HEV-1, HEV-5, and HEV-7, do not infect these animals. Our research group’s earlier investigations demonstrated that Mongolian gerbils (*Meriones unguiculatus*) are broadly susceptible to HEV [21]. Although the sensitivity depended on the genotype, we observed that HEV-1, -4, -5, and -8, and rat HEV replicate in *M. unguiculatus*, thus providing a useful small-animal model for these HEVs. For example, using Mongolian gerbils as animal models, we demonstrated that open reading frame 4 (ORF 4) is not essential in the replication and infection of HEV-1 [22]. A gerbil-adapted strain that induces a robust, acute HEV infection in gerbils has also been reported [23].

HEV-3 is also responsible for major zoonotic HEV infections, and a small-animal model for HEV-3 is also needed. Mongolian gerbils have shown poor susceptibility to HEV-3 [21], and more information about HEV-3 and characterization of the strains included in this genotype are desired. HEV-3 is genetically diverse and contains at least 15 subtypes (HEV-3a to -3o) and the subtype HEV-3ra [24,25]. Recently, the HEV-3a Kernow C1 strain (HQ389544) has been confirmed to infect Mongolian gerbils [26]. We speculated that it would be informative to determine whether the subtypes of HEV-3 show differing susceptibility in Mongolian gerbils. In the present study, we used HEV-3b, -3e, -3f, -3k, and -3ra to inject Mongolian gerbils, and we then compared the strains’ infectivity to determine whether the gerbils are useful as a small-animal model for these HEV-3 strains.

## 2. Materials and Methods

### 2.1. HEV Strains

Six HEV-3 strains from five subtypes were used in this study (Table 1): HEV-3b/wb (WB0567c1, LC774371); HEV3b/ch (HEV-3b-Chiba, LC786331); HEV-3e (WA1543, JQ026407); HEV-3f (JAO-SpaTok12, LC055972); HEV-3k (HEV83-2-27, AB740232); and HEV-3ra (JP-59, LC535077).

Strain HEV-3b/wb was obtained from a wild boar in Japan, and strain HEV-3k was derived from a pig fecal specimen collected from a piggery in Japan. Both strains were isolated by cell culture with a human hepatocarcinoma cell line, PLC/PRF/5 (JCRB0406) [21,27]. Strain HEV-3b/ch and strain HEV-3f were produced by a reverse genetics system using PLC/PRF/5 cells. Strains HEV-3b/ch, HEV-3b/wb, HEV-3f, and HEV-3k were grown in PLC/PRF/5 cells, and the cell culture supernatants were used for injection. HEV-3e was first detected in a Japanese monkey with persistent infection [28], and HEV-3ra was isolated from a feral rabbit by transmitting it to a Japanese white rabbit [29]. Fecal specimens collected from the above-mentioned Japanese monkey or Japanese white rabbit were used for injection in the present study. The culture supernatants or 10% fecal suspensions were centrifuged at 10,000× *g* for 30 min and passed through a 0.45-µm membrane filter (Millipore, Bedford, MA, USA). All samples were adjusted to contain viral RNA copy numbers of 10^7^ copies/mL and were stored at −80 °C.

We also used three strains derived from other genotypes: HEV-4i (HEV121-12, LC657084); HEV-5 (JBOAR135-Shiz09, AB573435); and rat HEV (V-105, JX120573). Mongolian gerbils were experimentally infected with these viruses through an intraperitoneal injection, and then, fecal specimens were collected, and the 10% suspensions were clarified as described above [21].

### 2.2. Injection of Mongolian Gerbils and the Sample Collection

Six-week-old female Mongolian gerbils (MON/Jms/GbsSlc, SLC, Hamamatsu, Japan) were randomly separated into six groups (*n* = 5 per group). Based on the results of our previous studies, each group was intraperitoneally injected with 1 mL (10^7^ copies/mL) of the virus solution. The fecal specimens were collected on days 4, 7, 11, 14, 18, 21, 25, and 28 post-injection (p.i.), and the 10% fecal suspensions were used to detect the viral RNA. At the end of each experiment, the gerbils were euthanized by exsanguination from the heart under anesthesia, and the liver, spleen, bile, and serum were collected. Tissues of the liver and spleen were washed three times with phosphate-buffered saline (PBS) and homogenized with the use of a MagNA Lyser (Roche, Mannheim, Germany) according to the manufacturer’s recommendations to prepare the 10% (*w*/*v*) tissue suspensions.

The animal experiments were reviewed and approved by the institutional ethics committee of Japan’s National Institute of Infectious Diseases (NIID) and performed according to the Guides for Animal Experiments issued by the NIID under code 123023 (21 May 2023). All of the gerbils were negative for the serum anti-HEV IgG antibodies in an enzyme-linked immunosorbent assay (ELISA) and negative for HEV RNA as confirmed by real-time reverse transcription–quantitative polymerase chain reaction (RT-qPCR) using the fecal specimens before the injection experiments. The ELISA and RT-qPCR are described below. The gerbils were individually housed in a Biosafety Level-2 facility.

### 2.3. Extraction and Detection of HEV RNA

Extraction of viral RNA was carried out by a MagNA Pure 96 System (Roche Applied Science, Mannheim, Germany) with a MagNA Pure 96 DNA and Viral NA Small Volume Kit (Roche Applied Science) from 200 µL of the samples.

The copy numbers of Viral RNA were examined by a one-step RT-qPCR using TaqMan Fast Virus 1-step Master Mix (Applied Biosystems, Foster City, CA, USA) and a QuantStudio 3 Real-Time PCR System (Applied Biosystems). The RT-qPCR was carried out under the condition of 5 min at 50 °C, 20 s incubation at 95 °C, followed by 40 cycles of 3 s at 95 °C and 30 s at 60 °C. A forward primer JVHEVF (5′-GGTGGTTTCTGGGGTGAC-3′ nt 5346–5363), a reverse primer JVHEVR (5′-AGGGGTTGGTTGGATGAA-3′ nt 5393–5415), and a probe JVHEVP (5′-FAM-TGATTCTCAGCCCTTCGC-TAMRA-3′ nt 5369–5386) were used to determine the RNA copy numbers of HEV-3, HEV-4, and HEV-5 [30]. A forward primer 5′-CCACGGGGGTTAATACTGC-3′ (nt 36–54), a reverse primer 5′-CGGATGCGACCAAGAAACAG-3′ (nt 189–208), and a probe 5′-FAM-CGGCTACCGCCTTTGCTAATGC-TAMRA-3′ (nt 81–102) were used to detect rat HEV RNA [14]. A 10-fold serial dilution of HEV-3 or rat HEV RNA (10^1^–10^7^ copies) was used as the standard to quantify the copy numbers [31]. Amplification data were collected and analyzed with Sequence Detector software ver. 1.3 (Applied Biosystems).

### 2.4. Detection of Anti-HEV IgG Antibodies

Serum anti-HEV IgG antibodies were detected by an ELISA using virus-like particles (VLPs) as the antigen as described with slight modification [32,33]. Flat-bottomed 96-well polystyrene microplates (Immulon 2, Dynex Technologies, Chantilly, VA, USA) were coated with the VLPs (100 ng/well): VLPs of HEV-1 were used to detect anti-HEV-3, HEV-4i, and HEV-5 IgG antibodies, and VLPs of rat HEV were used to detect the anti-rat HEV IgG antibodies. Duplicates of the serum samples (1:200) were examined.

Rabbit anti-Mongolian gerbil IgG antibody (H + L) conjugated with horseradish peroxidase (HRP) (1:1000) (Bioss, Boston, MA, USA) was used as the secondary antibody. The cut-off value of the anti-HEV IgG antibodies was 0.15, as described [21]. For the measurement of the anti-HEV IgG antibody titers, the serum samples were subjected to two-fold dilution starting from 1:200, and the highest dilution that showed a positive result was considered the antibody titer.

### 2.5. Determination of ALT Levels

The activities of the liver enzyme alanine transaminase (ALT) in the gerbil sera were measured using a Fuji Dri-Chem Slide GPT/ALT-PIII kit (Fujifilm, Saitama, Japan). We considered the gerbils’ pre-injection mean ALT value the normal value, and a two-fold or greater increase was considered a sign of ALT elevation [21].

### 2.6. Histopathology

Liver tissues from HEV-infected Mongolian gerbils collected on day 28 p.i. were fixed in 10% phosphate-buffered formalin and routinely embedded in paraffin. Tissue sections were cut into 2-μm serial sections, stained with hematoxylin and eosin (H&E), and used for histopathological examination.

### 2.7. Statistical Analysis

Statistical analyses of viral RNA copy numbers (log_10_) and anti-HEV IgG antibody titers (log_2_) were performed using GraphPad Prism software ver. 10.1.2 (GraphPad, La Jolla, CA, USA). Data normality was confirmed using the Kolmogorov–Smirnov test, and the significance of differences was then examined by a one-way analysis of variance (ANOVA), followed by the Tukey–Kramer test. Probability (*p*)-values ≤ 0.05 were considered significant.

## 3. Results

### 3.1. The Gerbils Showed Different Susceptibilities That Were Dependent on the HEV-3 Strains and Subtypes

**Figure 1:**
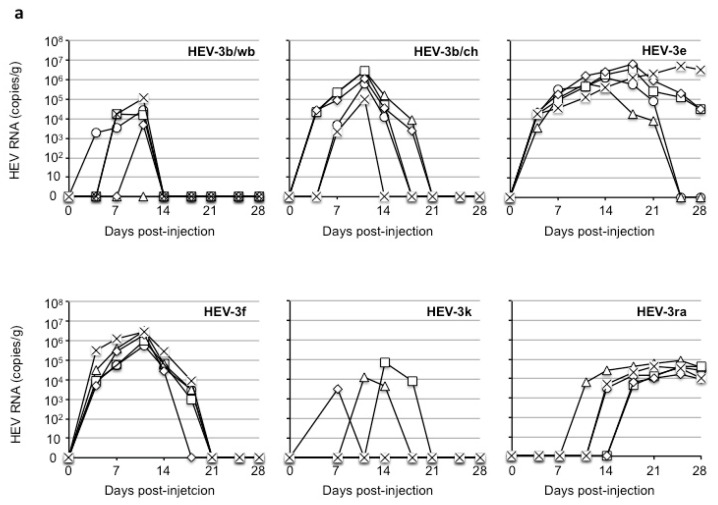

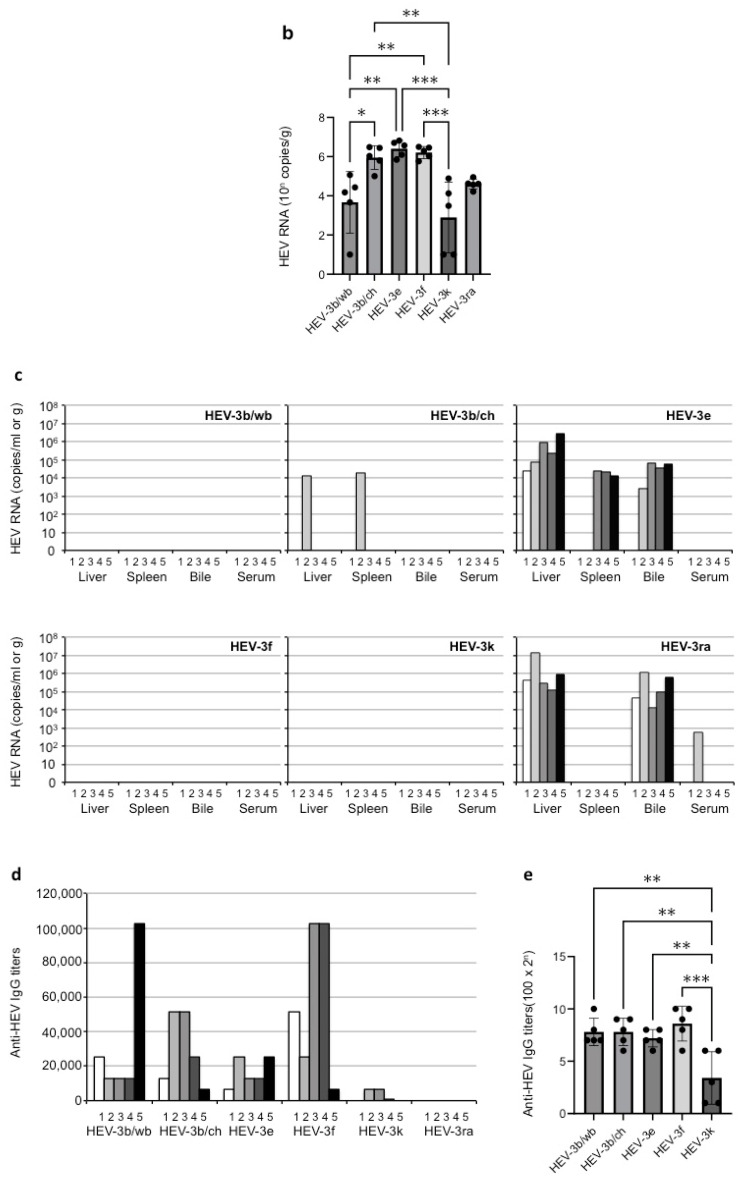
Replication of subtypes of HEV-3 in Mongolian gerbils. Thirty Mongolian gerbils were randomly separated into six groups (*n =* 5 per group). In each group, individual gerbils are numbered 1 to 5 and indicated by ○, △, ☐, ◇ and ✕ (**a**) or white, gray, or black bars as shown by 1 to 5 (**c**,**d**). Each group received 1 mL of the virus solution containing 10^7^ copies of the viral RNA derived from HEV-3b/wb, HEV-3b/ch, HEV-3e, HEV-3f, HEV-3k, or HEV-3ra via intraperitoneal injection. The fecal specimens were collected 2×/week, and serum, bile, liver, and spleen samples were collected at the end of the experiment (day 28 p.i.). The kinetics of the viral RNA in the fecal samples were measured by RT-qPCR (**a**). The statistical significance of the viral RNA titer in the peaks is shown (**b**). Bars: the average titers of viral RNA. Dots: individual RNA titers. * *p* < 0.05; ** *p* < 0.01; *** *p* < 0.001. The viral RNA in the liver, spleen, bile, and serum samples collected on day 28 p.i. was measured by RT-qPCR (**c**). The anti-HEV-IgG antibody titers were determined by an ELISA with the virus-like particles (VLPs) of HEV-1 as the antigens (**d**). The significance of the anti-HEV IgG antibody titers is also shown (**e**). Bars: the average titers of anti-HEV IgG antibody titers. Dots: individual antibody titers. ** *p* < 0.01; *** *p* < 0.001.

We randomly separated Mongolian gerbils into six groups (*n =* 5 per group) and intraperitoneally injected each gerbil with the virus solution containing 1.0 × 10^7^ copies/mL of the viral RNA derived from strain HEV-3b/wb, HEV-3b/ch, HEV-3e, HEV-3f, HEV-3k, or HEV-3ra. Fecal specimens were then collected 2×/week, and 10% of the specimens were used to detect the viral RNA by RT-qPCR. As shown in Figure 1a, the viral RNAs were detected in all of the HEV-3b/ch-, HEV-3e-, HEV-3f-, and HEV-3ra-injected gerbils. The viral RNA titers in the peaks were as follows: 1.3 × 10^6^ to 6.6 × 10^6^ copies/g in the HEV-3e-injected gerbils; 1.0 × 10^5^ to 3.0 × 10^6^ copies/g in the HEV-3b/ch-injected gerbils; 5.8 × 10^5^ to 3.1 × 10^6^ copies/g in the HEV-3f-injected gerbils; and 3.4 × 10^3^ to 8.9 × 10^4^ copies/g in the HEV-3ra-injected gerbils. Although the peak copy numbers detected in the HEV-3ra-injected gerbils were lower than those in the HEV-3b/ch-, HEV-3e-, and HEV-3f-injected gerbils, there was no significant difference, as confirmed by the statistical analysis (*p* > 0.10) (Figure 1b).

In all five of the HEV-3e-injected gerbils, the viral RNA was first detected on day 4 p.i. and remained detectable for over 18 days (from day 4 to day 21 p.i. for two gerbils, from day 4 to day 28 p.i. for the other gerbils). In contrast, in the HEV-3b/ch-injected gerbils, the viral RNA-positive period was <15 days (day 4 to day 18 p.i. for two gerbils, day 4 to day 18 p.i. for one gerbil, day 7 to day 18 p.i. for one gerbil, and day 7 to day 11 p.i. for one gerbil). In the HEV-3f-injected gerbils, the viral RNA-positive period was also <15 days (days 4–18 p.i. for four gerbils and days 4–14 p.i. for one gerbil) (Figure 1a). The viral RNA appeared later (after day 11, 14, or 18 p.i.) in the HEV-3ra-injected gerbils; however, the exact period of virus release was unknown since the experiments ended on day 28 p.i.

In contrast, the viral RNAs were detected from four of the five HEV-3b/wb-injected gerbils, and the copy numbers in the peaks were 4.7 × 10^3^ to 1.1 × 10^5^ copies/g, which is significantly lower than those in HEV-3b/ch-injected gerbils (*p* < 0.05), the HEV-3e-injected gerbils (*p* < 0.01), and the HEV-3f-injected gerbils (*p* < 0.01) (Figure 1a,b). Similarly, the viral RNAs were detected from three of the five HEV-3k-infected gerbils, and the copy numbers in the peaks were 3.1 × 10^3^ to 7.6 × 10^4^ copies/g, which is significantly lower than those in the HEV-3b/ch-injected gerbils (*p* < 0.01), HEV-3e-injected gerbils (*p* < 0.001), and HEV-3f-injected gerbils (*p* < 0.001) (Figure 1a,b). The detectable viral RNA in fecal specimens from HEV-3b/wb- and HEV-3k-injected gerbils was identified within 10 days (Figure 1a). These results suggest that HEV-3e, HEV-3f, and HEV-3b/ch were more efficiently replicated in gerbils compared to HEV-3e/wb and HEV-3k.

These results indicate that Mongolian gerbils are susceptible to HEV-3 strains, although their susceptibility does not seem to be the same among the HEV-3 subtypes, as reflected by the varying viral RNA titers and RNA-positive periods. In addition, the gerbils showed different susceptibility to two strains, HEV-3b/wb and HEV-3b/ch, although these strains belong to the same HEV-3b subtype.

All serum, bile, liver, and spleen samples were collected on day 28 p.i. The viral RNA was detected in all of the samples from HEV-3e-injected gerbils: liver (*n =* 5 samples); spleen (*n =* 3); and bile (*n =* 4) (Figure 1c). These results are similar to those obtained in our previous study in which the viral RNA was detected in the liver and spleen derived from HEV4i-, HEV-5-, and rat HEV-infected gerbils [21]. Although the viral RNA was detected in all five liver and bile samples and one serum sample from HEV-3ra-injected gerbils, it was undetectable in the spleens.

In contrast, viral RNA was not detected on day 28 p.i. in any of the samples collected from the gerbils injected with the HEV-3b/wb, HEV-3f, or HEV-3k strains, and it was detected in only one liver and one spleen sample from HEV-3b/ch-injected gerbils. These results suggest that transient infection occurred in these Mongolian gerbils with a short virus RNA-positive period. Here, too, the gerbils exhibited different sensitivities dependent on the HEV-3 subtype strains.

We next measured the anti-HEV IgG antibodies in the sera collected on day 28 p.i. by an ELISA as described in the Materials and Methods section. The antibodies were detected in all of the gerbils injected with HEV-3b/wb, HEV-3b/ch, HEV-3e, or HEV-3f (Figure 1d), and the titers were 1:6400–1:102,400 in the HEV-3b/wb group, 1:6400–1:51,200 in the HEV-3b/ch group, 1:6400–1:25,600 in the HEV-3e group, and 1:6400–1:102,400 in the HEV-3f group, with no significant differences among these groups (Figure 1e). In contrast, the antibodies were detected in only three HEV-3k-inoculated gerbils, and the titers were 1:800–1:6400, significantly lower than those in HEV-3b/wb-, HEV-3b/ch-, HEV-3e-, and HEV-3f-injected gerbils *(p <* 0.01) (Figure 1d,e).

Unexpectedly, although the viral RNA was detected in the feces (Figure 1a), liver, bile, and serum (Figure 1c) of these gerbils, all of the HEV-3ra-injected gerbils were negative for the anti-HEV IgG antibody (Figure 1d), suggesting that the replication of HEV-3ra in the gerbils induced a distinct immune response compared to that of the other HEV-3 subtypes.

Together, these findings indicate that Mongolian gerbils have broad but distinguishable susceptibility to HEV-3, depending on the subtype and strain. The viral RNA was detected in the feces (Figure 1a), and high titers of the serum anti-HEV IgG antibodies were induced in all of the HEV-3b/ch- and HEV-3e-injected gerbils and nearly all of the HEV-3b/wb and HEV-3f-injected gerbils (Figure 1d), suggesting that Mongolian gerbils had potential as small-animal models for HEV-3 research.

### 3.2. Replication of HEV-3e, -4i, -5, and Rat HEV in Mongolian Gerbils

To further examine the susceptibilities of the gerbils to HEV-3, we compared HEV-3e with three replication-competent HEV strains: HEV-4i; HEV-5; and rat HEV (a genotype HEV-C1 classified in the genus *Rokahepevirus*) (Table 1). A total of 20 gerbils were randomly separated into four groups (*n =* 5 per group). Each gerbil was intraperitoneally injected with 1.0 mL of the 10% fecal suspension containing 1.0 × 10^5^ copies/mL of the viral RNA. The fecal specimens were collected 2×/week, and 10% fecal suspensions were used to detect the viral RNA by RT-qPCR.

**Figure 2:**
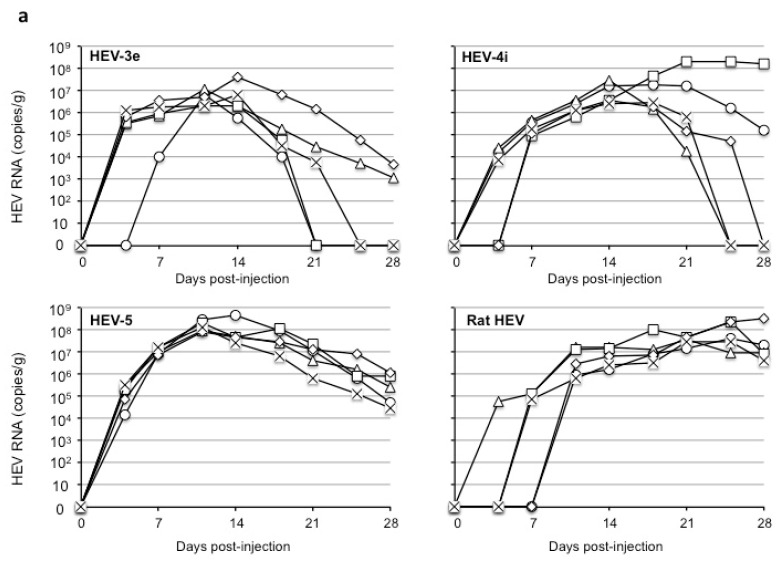

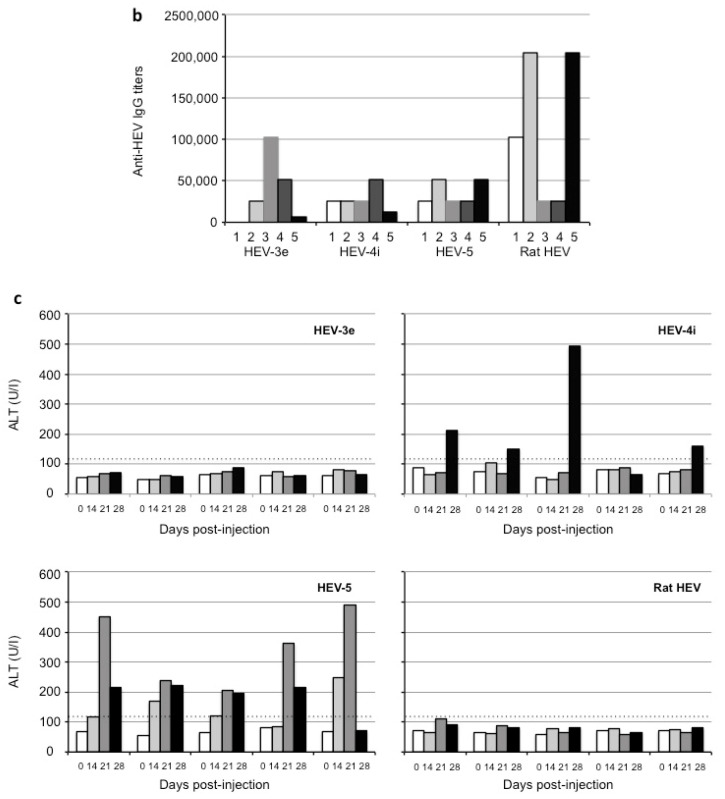
Replication of HEV-3e, HEV-4i, HEV-5, and rat HEV in Mongolian gerbils. Four groups of Mongolian gerbils (*n =* 5 per group) were injected with the 10% stool suspension prepared from HEV-3e-infected. Individual gerbils are numbered 1 to 5 and indicated by ○, △, ☐, ◇ and ✕ (**a**), or white, gray, and black bars (**b**,**c**). The fecal specimens were collected 2×/week, and the kinetics of the viral RNA were determined by RT-qPCR (**a**). The anti-HEV IgG titer in serum samples collected on day 28 p.i. was determined by an ELISA (**b**). The ALT values detected in the serum are shown; the numbers 0, 14, 21, and 28 indicate the serum collected on days 0, 14, 21, and 28 p.i., respectively (**c**). Dotted lines: Two-fold the normal ALT value (118 IU/L) (**c**).

Virus replication was observed in all 20 gerbils (Figure 2a). The RNA copy numbers in the peaks ranged from 2.2 × 10^6^ to 4.0 × 10^7^ copies/g in the HEV-3e-injected gerbils, 3.1 × 10^6^ to 2.2 × 10^8^ copies/g in the HEV-4i-injected gerbils, 8.2 × 10^7^ to 4.8 × 10^8^ copies/g in the HEV-5-injected gerbils, and 2.9 × 10^7^ to 3.5 × 10^8^ copies/g in the rat HEV-injected gerbils.

We used the serum samples collected on day 28 p.i. for the detection of anti-HEV IgG antibodies. The antibodies were detected in all of the HEV-infected gerbils with the exception of one HEV-3e-infected gerbil (Figure 2b). The IgG titers were 1:6400 to 1:102,400 in the HEV-3e-infected group, 1:12,800 to 1:51,200 in the HEV-4i-infected group, 1:25,600 to 1:51,200 in the HEV-5-infected group, and 1:25,600 to 1:204,800 in the rat HEV-infected group. These results demonstrate that although the viral RNA copy numbers in the HEV-3e-injected gerbils were slightly lower than those in the HEV-4i-, HEV-5-, and rat HEV-infected gerbils, the HEV-3e recovered from the gerbils’ fecal specimens was infectious, again indicating that Mongolian gerbils are susceptible to HEV-3e.

To determine whether the virus infection induced liver damage in the gerbils, we collected serum samples on days 0, 14, 21, and 28 p.i. and measured the ALT levels in the sera (Figure 2c). The ALT values in the 20 gerbils before injection ranged from 54 to 88 IU/L, with a mean value of 59 IU/L considered the normal ALT value. ALT values > 118 IU/L (i.e., a two-fold increase) were considered a sign of ALT elevation. The values in all specimens from the HEV-3e-infected and rat HEV-infected gerbils were <118 IU/L. In contrast, ALT elevation was observed in three of the five HEV-5-infected gerbils on day 14 p.i., ranging from 119 to 248 IU/L; in all five gerbils on day 21 p.i., ranging from 205 to 452 IU/L; and in four of the five gerbils on day 28 p.i., ranging from 197 to 221 IU/L.

Similarly, ALT values ranging from 149 to 492 IU/L were observed on day 28 p.i. in four of the five HEV-4i-infected gerbils. These results indicated that liver damage occurred in gerbils infected with strain HEV-4i or strain HEV-5 but not in those infected with HEV-3e or rat HEV, demonstrating that HEVs belonging to different genera or genotypes showed different pathogenicity in Mongolian gerbils.

### 3.3. Histopathological Lesions in Liver Samples from HEV-Infected Gerbils

**Figure 3:**
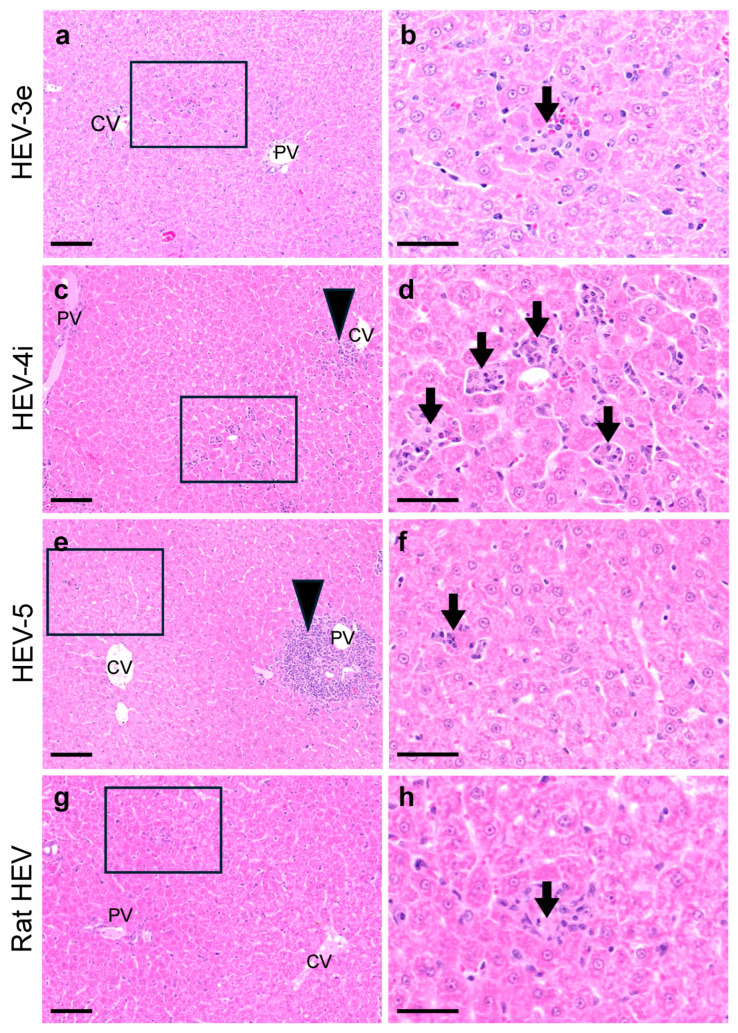
Histopathology of liver tissues from HEV-infected Mongolian gerbils. Representative H&E-stained liver sections from Mongolian gerbils infected with HEV-3e (**a**,**b**), HEV-4i (**c**,**d**), HEV-5 (**e**,**f**), or rat HEV (**g**,**h**) collected on day 28 p.i. are shown. Left column: photos of low-power fields (**a**,**c**,**e**,**g**); scale bars = 100 μm. Right column: photos of high-power fields (**b**,**d**,**f**,**h**); scale bars = 50 μm. Arrowheads: infiltration of inflammatory leukocytes. Arrows: foci of single-cell necrosis followed by phagocytosis by macrophages.

The histopathological examination of liver tissues from HEV-infected Mongolian gerbils collected on day 28 p.i. revealed infiltrations of lymphocytic inflammatory cells into portal areas in the gerbils infected with HEV-4i or HEV-5 (Figure 3c,e). Foci of single-cell necrosis were frequently observed in the HEV-4i-infected gerbils and occasionally noted in the HEV-5-infected gerbils (Figure 3d,f). In contrast, in the HEV-3e-infected gerbils, no clear infiltration of lymphocytes was observed, and only a few foci of single-cell necrosis appeared in the liver (Figure 3a,b). In the liver samples from rat HEV-infected gerbils, only mild lymphocyte infiltration and a few single-cell necrosis foci were observed (Figure 3g,h). Although the liver tissues were collected only on day 28 p.i., these findings suggested that histopathological lesions occurred at least in HEV-4i- and HEV-5-infected Mongolian gerbils.

## 4. Discussion

The infectivity of six strains from five HEV-3 subtypes in Mongolian gerbils was assessed in this study, and the results of our experiments confirmed that Mongolian gerbils were broadly susceptible to these strains. The infectivity was considerably different among the subtypes and even between strains in the same subtype, HEV-3b. The subtypes HEV-3, HEV-3b, HEV-3f, and especially HEV-3e replicated extensively in Mongolian gerbils, and they produced the viruses at high titers for an extended period. Together, these results demonstrate that Mongolian gerbils have potential as an animal model of HEV-3.

Our observations also confirmed that Mongolian gerbils are poorly susceptible to HEV-3k. Although the JP-59 strain of HEV-3ra was able to infect gerbils, the copy numbers of the viral RNA were low, and no anti-IgG antibody responses were induced by this strain, suggesting that the replication of HEV-3ra in the gerbils was unusual. The reasons for the poor replication of HEV-3ra in Mongolian gerbils are still unclear. Further studies need to focus on the genetic difference in the host–virus interaction. Since the genotype HEV-3 includes at least 16 subtypes, it is worth exploring whether other subtypes would be more efficiently replicated in Mongolian gerbils.

Our group’s earlier investigation confirmed that the rbIM223LR strain of HEV-3ra does not infect Mongolian gerbils after experimental inoculation [21]. However, the results of the present study confirmed that the JP-59 strain (which belongs to the same HEV-3ra subtype) infected Mongolian gerbils, again indicating that different strains in the same subtype, i.e., HEV-3ra, have different infectivity in Mongolian gerbils. The rbIM223LR strain was initially isolated from a farmed rabbit in Inner Mongolia, whereas the JP-59 strain was isolated from a feral rabbit in Japan; these strains share 87.4% of the nucleotide sequence identity [29,34]. The genetic basis underlying virus infectivity in gerbils is intriguing, and its clarification awaits further studies.

We also observed that two HEV-3b strains, HEV-3b/wb and HEV-3b/ch, exhibited a similar strain-specific difference. HEV-3b is a major subtype of HEV-3 that commonly circulates in pigs and wild boars and has caused hepatitis E in humans in Japan [27,35]. A comparison of the two strains’ nucleotide sequences revealed that the strains share 87.8% identity, suggesting that genetic diversity is common in the subtype HEV-3b. Taking our present results and the past findings together, we conclude that the infectivity of HEV in Mongolian gerbils depends not only on the subtypes but also on the strains.

Our above-cited investigation confirmed that Mongolian gerbils are a suitable small-animal model for evaluating the infectivity of HEV [21], but it was not clear whether HEV-infected gerbils exhibited liver damage. In the present study, we analyzed serum samples collected from HEV-3e-, HEV-4i, HEV-5-, and rat HEV-infected gerbils, and we observed that ALT elevation occurred only in HEV-4i-infected and HEV-5- infected gerbils (Figure 2c). Histopathological lesions were also observed in the livers of HEV-4i- and HEV-5-infected Mongolian gerbils (Figure 3). These results demonstrated that (i) the pathogenicity of HEVs in the gerbils also depended on the HEV genotype, and (ii) the HEV-4i-infected Mongolian gerbils and HEV-5-infected Mongolian gerbils might be a candidate animal model to examine the pathogenicity of HEV. Together, these findings suggest that Mongolian gerbils could be a convenient small-animal model that would be useful not only to elucidate the mechanisms of HEV replication but also to evaluate the efficacy of vaccines against HEV.

## Figures and Tables

**Table 1:** The hepatitis E virus (HEV) strains used in the present study.

Family	Subfamily	Genus	Species	Genotype	Subtype	Strain and Accession No.
*Hepeviridae*	*Orthohepevirinae*	*Paslahepevirus*	*balayani*	HEV-3	3b	HEV-3b/wb (WB0567c1. LC774371)
						HEV-3b/ch (HEV-3b-Chiba, LC786331)
					3e	HEV-3e (WA1543, JQ026407)
					3f	HEV-3f (JAO-SpaTok12, LC055972)
					3k	HEV-3k (G3HEV83-2-27, AB740232)
					3ra	HEV-3ra (JP-59, LC535077)
				HEV-4	4i	HEV-4i (G4HEV121-12, LC657084)
				HEV-5		HEV-5 (JBORA135-Shiz09, AB573435)
		*Rocahepevirus*	*ratti*	HEV-C1		Rat HEV (V-105, JX120573)

## Data Availability

The sequences of HEV used in this study have been assigned (GenBank accession no. LC774371, LC786331, JQ026407, LC055972, AB740232, LC535077, LC657084, AB573435, and JX120573).
